# OpenClinica

**DOI:** 10.1186/2043-9113-5-S1-S2

**Published:** 2015-05-22

**Authors:** Marinel Cavelaars, Jacob Rousseau, Cuneyt Parlayan, Sander de Ridder, Annemarie Verburg, Ruud Ross, Gerben Rienk Visser, Annelies Rotte, Rita Azevedo, Jan-Willem Boiten, Gerrit A Meijer, Jeroen A M Belien, Henk Verheul

**Affiliations:** 1 The Hyve, 3527 KT Utrecht, the Netherlands; 2 Dept of Medical Oncology, VU Medical Center Amsterdam, 1081 HV Amsterdam, the Netherlands; 3 Dept of Pathology, VU Medical Center Amsterdam, 1081 HV Amsterdam, the Netherlands; 4 Clinical Research Bureau, VU Medical Center Amsterdam, 1081 HV Amsterdam, the Netherlands; 5 Netherlands eScience Center, Science Park, 1098 XG Amsterdam, Amsterdam, the Netherlands; 6 TrialDataSolutions, Amsterdam, the Netherlands; 7 Center for Translational Molecular Medicine, High Tech Campus, 5656 AG Eindhoven, the Netherlands

## Characterisation

Software tool, CDMS (clinical data management system), clinical trials, eCRF, open source community edition.

## Tool description

OpenClinica is an electronic Case Report Form (eCRF) tool designed to capture clinical trial data. The tool is web-based and thus can be accessed from most locations in the world to support multi-center studies. It is a ‘do-it-yourself’ tool: users can operate all the modules and functions that are described below and system administrators are not required to perform study specific actions [1].

OpenClinica is an open source tool and has two editions: Community and Enterprise edition. The OpenClinica Community edition is freely available, while the Enterprise edition has additional capabilities, is commercially supported and is therefore subject to a license fee. The CTMM-TraIT (Translational Research IT) project aims to establish a long-lasting IT infrastructure for translational biomedical research in the Netherlands and hosts the OpenClinica Community edition. The CTMM-TraIT OpenClinica server is currently used for data capture in 120 clinical studies involving 600 users from 146 different centers.

**Figure 1:**
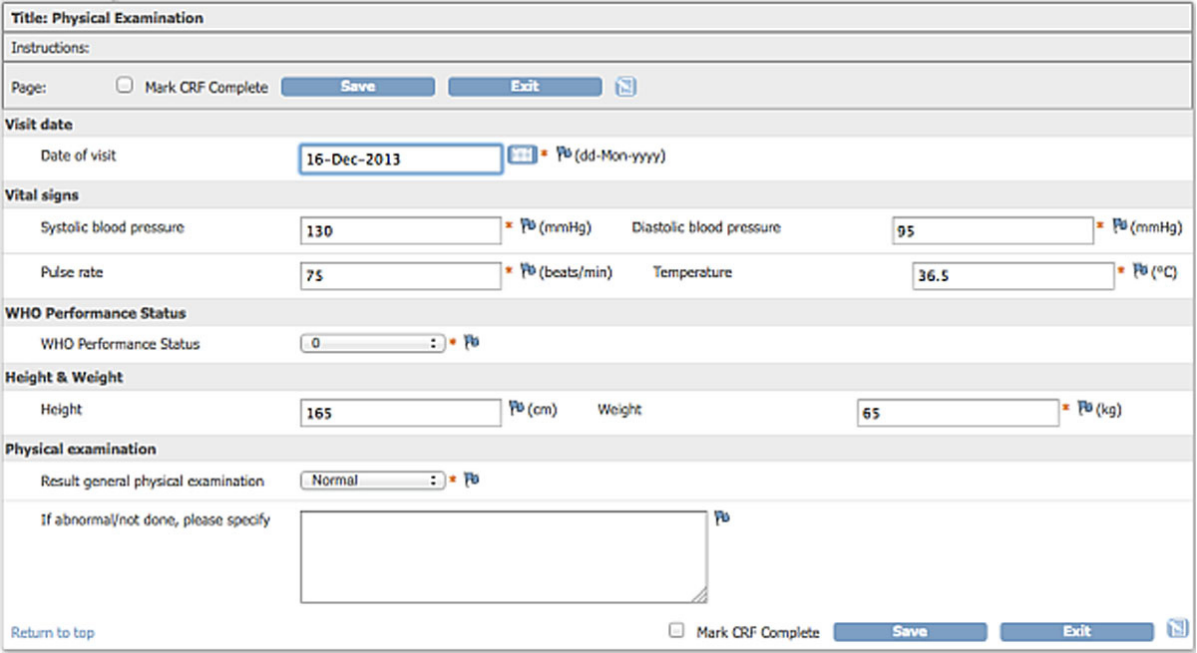
Example of a completed Case Report Form (CRF) in OpenClinica (systolic blood pressure, pulse, etc.)

OpenClinica has a modular design with separate modules for study setup, data submission, data monitoring and data extraction. The study setup module allows building CRFs (Figure 1), creating automated validation checks and creating sites. Most people are able to build a study, including the more advanced CRF functions, after only two days of training. Data can be submitted to the CRF by manual entry or by uploading, either via the user interface or via the OpenClinica web services. With the Monitor and Manage Data module, data for a study can be overseen and validated. The module consists of Source Data Verification features to track user evaluations. The Study Audit Log allows viewing the change history of entered data for each study subject. The Notes and Discrepancies module provides a means for users to document, communicate, and manage issues about data in a clinical trial. The Extract Data module allows easy exporting of the collected clinical trials data in different formats (CDISC ODM XML, HTML, Excel, SPSS and Tab-delimited text). OpenClinica works with controlled access through user accounts and authorizations.

## Status of development

OpenClinica Community Version: 3.4 (October 2014).

## Users

A large international user community exists (clinical researchers, data managers)

## Links

[https://community.openclinica.com] and OpenClinica user documentation [https://docs.openclinica.com]
